# Comparative proteomic analysis of genetically modified maize grown under different agroecosystems conditions in Brazil

**DOI:** 10.1186/1477-5956-11-46

**Published:** 2013-12-04

**Authors:** Sarah Zanon Agapito-Tenfen, Miguel Pedro Guerra, Odd-Gunnar Wikmark, Rubens Onofre Nodari

**Affiliations:** 1 CropScience Department, Federal University of Santa Catarina, Road Admar Gonzaga 1346, Florianópolis 88034-000 Brazil; 2 Genøk, Center for Biosafety, The Science Park, P.O. Box 6418 Tromsø 9294, Norway

**Keywords:** Profiling techniques, GMO, Transgenic, Two-dimensional gel electrophoresis, Proteome

## Abstract

**Background:**

Profiling technologies allow the simultaneous measurement and comparison of thousands of cell components without prior knowledge of their identity. In the present study, we used two-dimensional gel electrophoresis combined with mass spectrometry to evaluate protein expression of Brazilian genetically modified maize hybrid grown under different agroecosystems conditions. To this effect, leaf samples were subjected to comparative analysis using the near-isogenic non-GM hybrid as the comparator.

**Results:**

In the first stage of the analysis, the main sources of variation in the dataset were identified by using Principal Components Analysis which correlated most of the variation to the different agroecosystems conditions. Comparative analysis within each field revealed a total of thirty two differentially expressed proteins between GM and non-GM samples that were identified and their molecular functions were mainly assigned to carbohydrate and energy metabolism, genetic information processing and stress response.

**Conclusions:**

To the best of our knowledge this study represents the first evidence of protein identities with differentially expressed isoforms in Brazilian MON810 genetic background hybrid grown under field conditions. As global databases on outputs from “omics” analysis become available, these could provide a highly desirable benchmark for safety assessments.

## Background

Genetically modified organisms (GMO) are widely grown and consumed in a number of countries. According to the industry agency, The International Service for the Acquisition of Agri-biotech Applications, biotech crops reached 170 million hectares planted with transgenic soybeans, maize and cotton in 2012 [1].

Despite the widespread use of GMOs by many countries, the need for biosafety research remains a concern [2]. Unfortunately, lack of stringent standards, international harmonization, and transparency, as well as remaining claims of confidentiality on biosafety-relevant data places additional burdens on regulatory agencies [3].

There is also a clear indication of a rapid evolution in the analytical tools used to assess both risks and potential benefits within the food chain, in particular, there is a growing focus on the development of high-throughput, non-targeted and broad scale approaches [4].

Evaluations of possible unintended effects derived from genetically modified crops by the use of proteomics and transcriptomics have already been reported [5-7]. Profiling technologies, such as proteomics, allow the simultaneous measurement and comparison of thousands of plant components without prior knowledge of their identity [8]. Thus, the combination of non-targeted methods facilitates a more comprehensive approach than targeted methods and thus provides additional opportunities to identify unintended effects of the genetic modification [9].

In addition, it is widely accepted that environmental conditions may cause considerable changes and responses in plants [10]. Field grown, transgenic MON810 maize, as any other crop, is inevitably subject to diverse environmental conditions and agricultural practices. Insight into the natural variation in gene expression has emerged as an important issue with respect to interpreting results from “omics” experiments and it should be investigated in plants grown in for instance, different locations, climates, years of harvest, and under different farming practices, to make this overview as complete as possible [11]. Comparison of transgenic and closely related hybrids under a set of variable environmental and cultural conditions is, therefore, highly desirable.

However, proteomics and other untargeted profiling techniques have been frequently criticized in the context of GM plant risk assessments as producing data which are difficult to interpret and that conventional breeding and environment introduce more quantitative variation than does genetic engineering [12].

In the present study, we used a proteomic approach to evaluate protein expression of GM maize hybrid grown under different agroecosystems conditions in Brazil. We have used MON810 maize hybrids widely grown by Brazilian farmers. Hybrid plants were field grown in two locations (Campos Novos and Chapecó), during one growing season.

## Results and discussion

In this study, we evaluated the protein expression profile of field-grown genetically modified maize expressing insecticide protein. To evaluate this, one GM P32R48YG maize hybrid (MON810 event; Yieldgard ®), containing a single insert, was subjected to comparative profiling using the near-isogenic non-GM hybrid P32R48 as the comparator. These hybrids were compared following cultivation at two locations (Campos Novos and Chapecó), during one growing season.

### Principal component analysis

Principal Component Analysis (PCA) was used to demonstrate similarities in the protein quantity between different gels. We have performed three PCA assessments: (i) GM and non-GM gels from the same location were first analyzed to characterize biological and technical variation, (ii) GM gels from the two locations were then analyzed to detect environmental influence, and the same was made for non-GM gels. Lastly, (iii) all gels were analyzed together to gain insight into possible genotype x environment interactions in the dataset. It is relevant to mention that each 2-DE run consisted of three biological samples from GM plants and three biological samples from non-GM plants from the same location. Therefore, when comparing gels from different locations, some technical variation could be hidden within other sources of variation.

**Figure 1:**
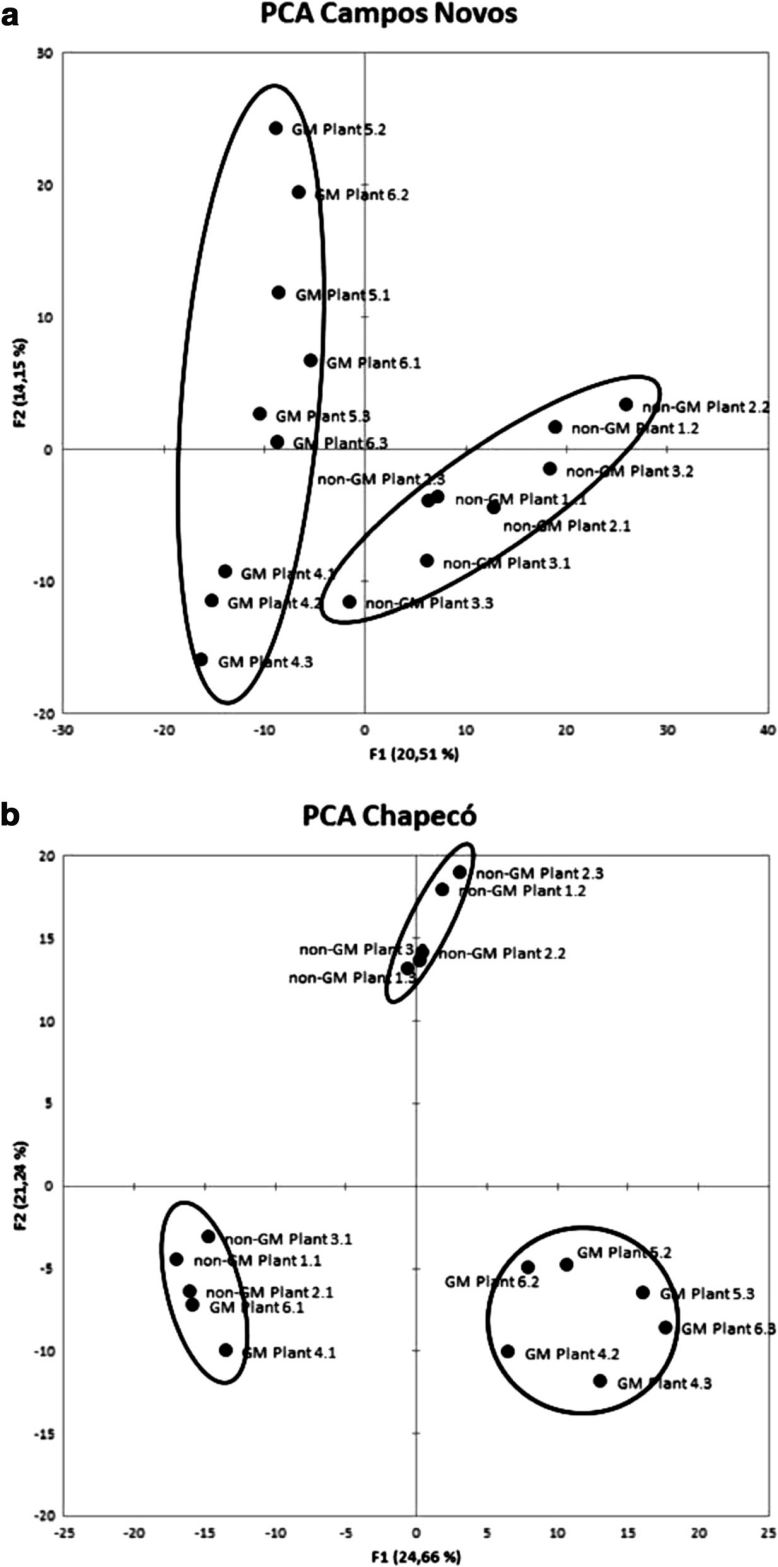
PCA score plots of genetically modified and non-genetically modified near isogenic maize hybrids grown at Campos Novos (Brazil) (a) and Chapecó (b), Brazil.

In the analysis of Campos Novos (Figure 1a), the first two eigenvalues corresponded to a 34.65% of the total variance in the dataset. A clear separation was seen between the GM and non-GM plants in the first factor of the PCA, which explained 20.5% of the total variation (F1 values below −15 and above +20, respectively). There was also variation within biological and technical replicates within each genotype (GM and non-GM), explaining 14.15%. Since 34.65% of the variation does not represent a high percentage of the total variation, careful must be taken when interpreting these results because other sources of variation might be present in the next factors.

At Chapecó (Figure 1b), one technical repetition seems to skew the plot towards the left, thus explaining 24.66% of the variation. This indicates that the protein patterns were somewhat different in this particular run. The between treatments (GM and non-GM) variation was also significant (21.24%) (F1 values above +10 and below −5, respectively). Again, the first two factors accounted for only 45.9% of the total variation.

In a recent review, Rabilloud et al. [13] have observed that all modern detection methods show a modal coefficient of variation (CV) of ca. 20% (including the variation of the 2-DE gel process). In addition, some other similar studies, such as Coll et al. [7] have also experienced variability between replicate gels from proteomic analysis of GM maize which felt in the range of 15%. Barros et al. [6] found up to 31% of variation explaining biological and technical variability within gels from the same GM maize line but no specific comparison was performed to differentiate these two sources of variation.

**Figure 2:**
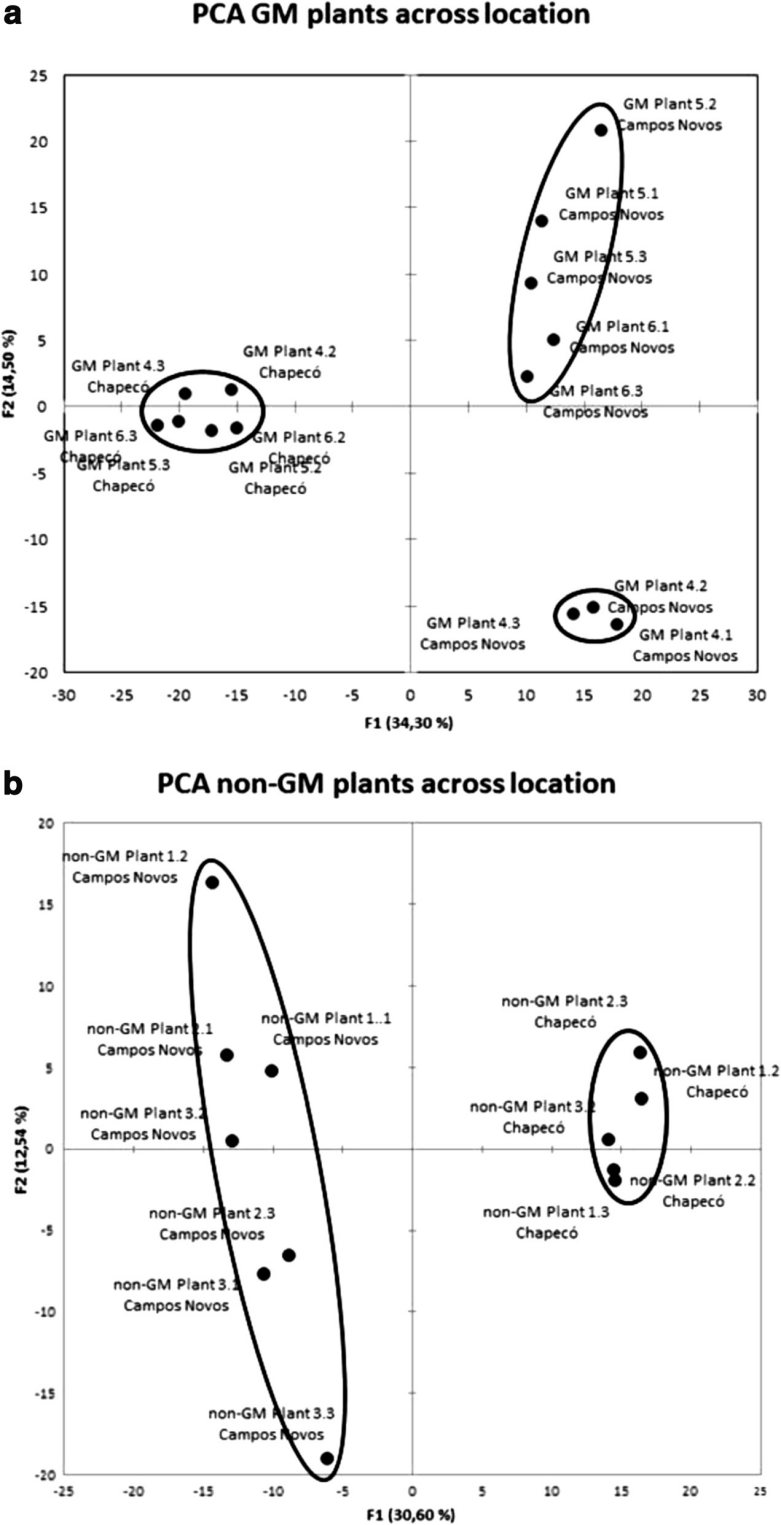
PCA score plots of genetically modified (a) and non-genetically modified near isogenic maize hybrids (b) across location (grown at Campos Novos and Chapecó, Brazil).

Our second analysis included the comparison between the same genotype across the two growing agroecosystems. When analyzing non-GM plants (Figure 2a), the major source of variation (30.6%) was explained by the different growing environments. Biological and technical variation pattern was also observed for non-GM gels from Campos Novos (12.54%). As for the analysis of GM gels, a clear separation was observed between GM plants grown at Campos Novos from those grown at Chapecó (Figure 2b), which explained 34.3% of the total variation in the dataset. In the second factor, some biological and technical variation explained 14.5% among GM gels from Campos Novos.

In terms of quantitative variation, we can conclude that the main sources of variation were the environment, the genotype, and variation deriving from biological and technical repetitions. Note that we have discarded one technical repetition from Chapecó samples, which has clearly shown an unexpected deviation. Taken together, these results show the relevance of detecting major sources of variation in the experiment dataset. Thus, for benchmarking and comparative analysis approaches, the deployment of broader scale, less biased analytical approaches for GM safety assessment should also embrace the issues of sources and extents of variation [4].

**Figure 3:**
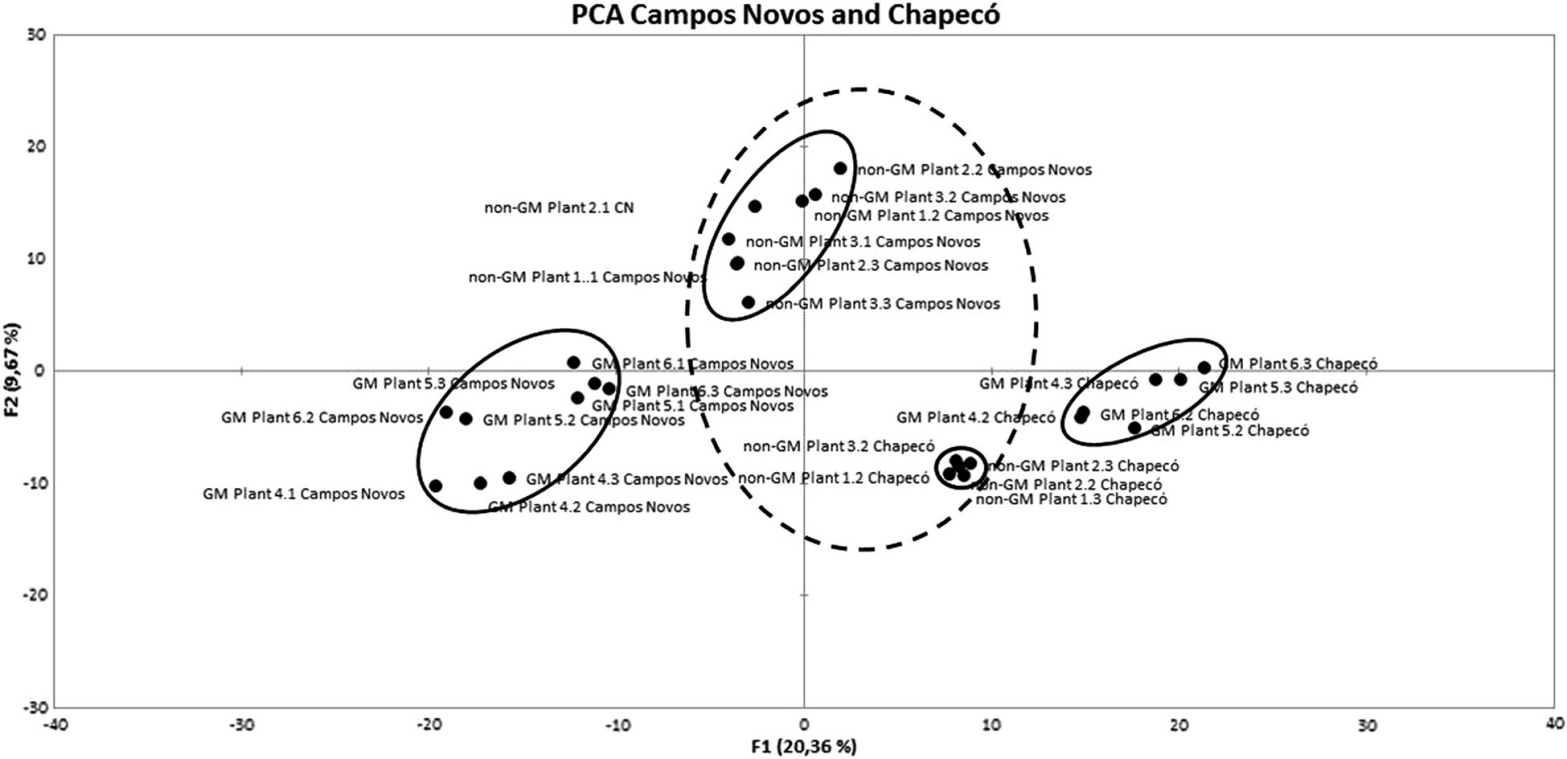
**PCA score plots of genetically modified and non-genetically modified near isogenic maize hybrids across both location (grown at Campos Novos and Chapecó, Brazil).** All gels from the experiment dataset were analyzed together.

When all gels are plotted together, an interesting pattern becomes evident (Figure 3). Although the first factor does separate Chapecó samples from samples from the other agroecosystem, Non-GM plants from Chapecó and Campos Novos are very close to the center of the plot (dashed round). In contrast, the GM hybrid proteomes from Chapecó are very distant from the proteomes of the same plants grown in Campos Novos. Consequently, gene expression profiles of the GM plants seem to be more affected by the environment.

It is well known that the way in which organisms react to different environments is as much part of its characteristics as its appearance and qualities in a single environment. At the present time, there is a great deal of interest in the way in which an individual can maintain stability in the face of varying environmental influences [14]. Zeller et al. [15] have used GM wheat variety carrying powdery mildew resistance gene as a model system to study potential transgene x environment interactions in genetically modified plants. These authors have observed that transgenes can have large effects on the entire phenotype of a plant and that these effects can sometimes be reversed when plants are moved from the glasshouse to the field. Nevertheless, it is unclear what are the mechanisms underlying these effects and how they may affect concepts in molecular plant breeding and plant evolutionary ecology [15].

### Proteomic profile of field grown GM maize

We have chosen two different agroecosystems for growing maize under field conditions. Although identical crop management has been used, climate and soil characteristics are distinct for these two locations in South Brazil.

The proteomic profiles using 2-DE was determined by the use of three technical replicates and coomassie blue protein staining. Quantitative protein differences between a GM hybrid and its comparable near-isogenic non-transgenic hybrid were investigated by comparison of nine 2-DE gels per treatment. Experimental variations have been avoided by the exclusion of spots that were not present in at least three gels within each treatment.

The total protein content mean was 1.84 ±0.04 mg.g^-1^ of fresh weight (Table 1). No statistically significant difference was found between treatment within each location and between locations. The average number of spots (520) on the 2-DE gel from GM and non-GM plants grown under Campos Novos and Chapecó field conditions showed similar patterns and they were considered well resolved for a 13 cm gel stained with coomassie blue. No statistically significant difference was found between treatments within each location. However, statistically significance difference was found between locations for the total number of spots detected (Table 1). It is important to mention that a direct comparison of proteome profiles between locations was not performed.

**Table 1:** Total protein content and number of detected spots on genetically modified hybrid (P32R48YG) and un-modified (P32R48) maize hybrid grown under farm conditions in Campos Novos and Chapecó, Brazil

**Location**	**Hybrid**	**Total protein content (mg.g**^**-1 **^**of fresh weight)**^**a**^	**Average number of detected spots**^**b**^	**ANOVA ( *P *)**
Campos Novos	Non-GM	2.03 ±0.19	617 ± 50	
	GM	2.01 ±0.26	643 ± 60	
	Mean value	2.02 ±0.01	630 ± 55	0,335
Chapecó	Non-GM	1.72 ±0.15	458 ± 73	
	GM	1.63 ±0.08	474 ± 42	
	Mean value	1.67 ±0.07	466 ± 58	0,585
	Mean value	1.84 ±0.04	520 ± 118^c^	<0,0001

^a^ Values are means of n = 9 samples ± standard deviation; ^b^ Values are means of n = 9 gels ± standard deviation; ^c^ Statistically significant.

### Mass spectral identification of differentially expressed proteins

**Figure 4:**
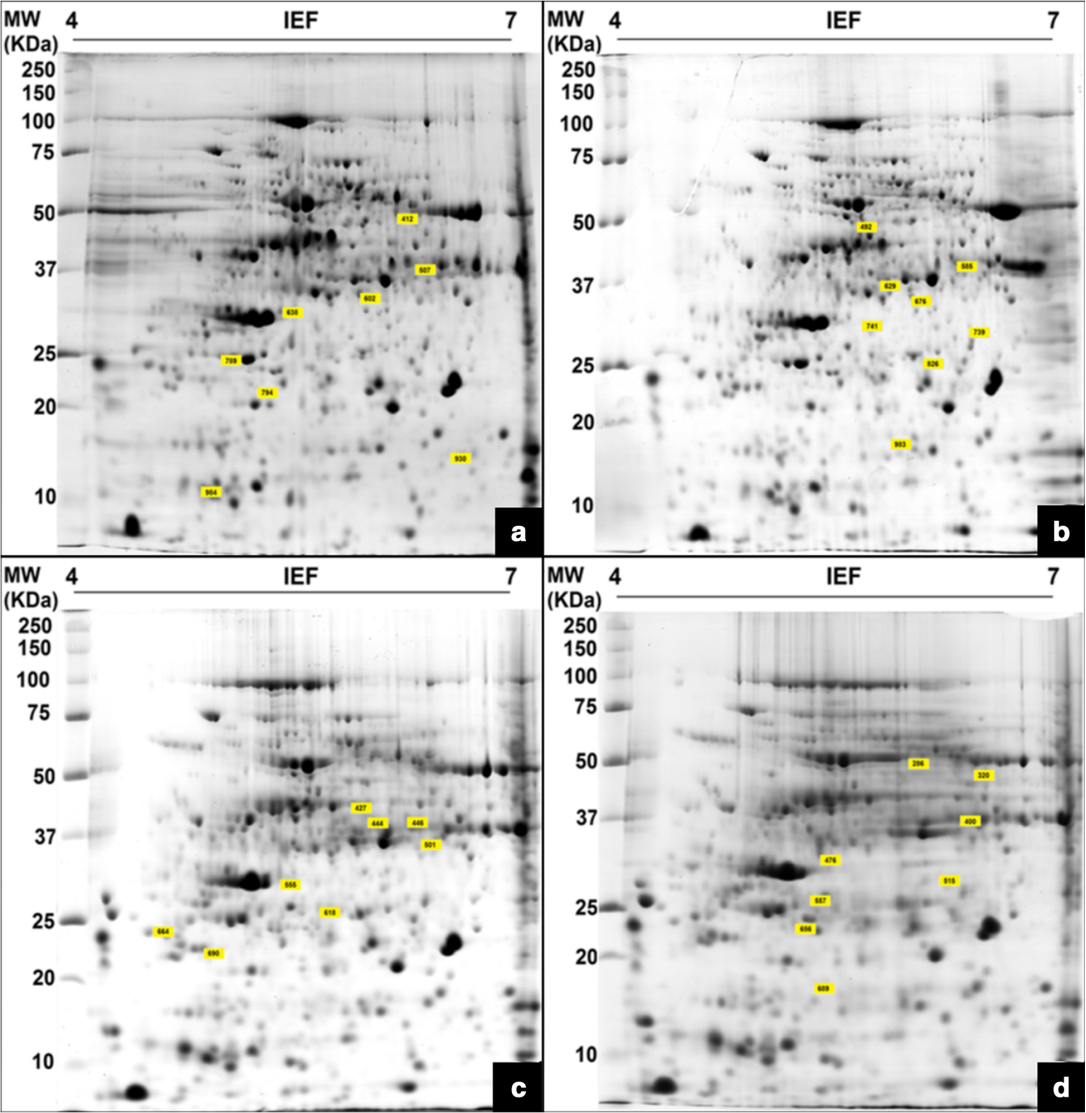
**Representative two-dimensional gel electrophoresis (2-DE) maps of the proteome of genetically modified maize plants (MON810 event) and non-modified maize grown in Campos Novos (4a and 4b respectively) and Chapecó (4c and 4d respectively) between pH 4 and 7.***Linear isoelectric focusing pH 4–7 for the first dimension and 12% SDS–PAGE gels in the second dimension were used. Molecular masses range from 10 to 250 kDa are given on the left side.* The top-left corners of the yellow boxes point to differentially expressed protein spots selected for mass spectrometry identification. ID of identified proteins from Table 2 are indicated in yellow boxes.

Comparison of the GM and non-GM plants revealed a total of 32 different proteins that were either present, absent, up- or down-regulated in one of the hybrids, at a statistically significant level (*P* < 0.05) (Figure 4). Proteins that were not detected in this study, they were either not present or below the detection limit of approximately 1 ng. These were then considered absent in the sample.

All 32 proteins were identified with Mascot scores value greater than 325 using Quadrupole Time-of-Flight (Q-TOF) tandem mass spectrometry analysis (MS/MS) (*P* < 0.05). These proteins were all identified in *Zea mays* species, with the exception of two proteins that were identified in *Hordeum vulgare* (barley) and *Brachypodium distachyon* (purple false brom; model grass for genetic studies that had its genome sequenced). Table 2 presents the MS/MS parameters and protein identification characteristics for both experiments. It was found that 16 proteins had differential expression levels in Campos Novos and the same number was found for Chapecó. Five of these proteins could not be characterized due to the lack of protein annotations in available databases.

**Table 2:** Differentially expressed proteins in P32R48 maize hybrid from control (non-genetically modified) and genetically modified P32R48YG (MON810) maize hybrid grown under two different agroecosystems (Campos Novos and Chapecó) in Brazil

**Spot**	**Fold variation**^**a**^	**Mascot score**	**Protein name**^**b**^	**Matched pep**	**NCBI Accession**	**Theor. MW**	**Theor. PI**	**Exp. MW**	**Exp. PI**	**Celullar component**	**Function**^**c**^	**Category**
**Campos Novos**												
492	Non-GM	1337	Adp-glucose pyrophosphorylase small subunit [zea mays]	14	gi|14582768	56	6.48	50	5.47	Amiloplast, apoplast, chloroplast, cytosol	Starch biosyntesis (nucleotydiltransferase)	Carbohidrate metabolism
629	Non-GM	848	Fructose-bisphosphate aldolase [zea mays]*	10	gi|223975775	38	6.37	38	5.65	Cell wall, chloroplast, cytosol, mitochondrion, nucleolus, membrane	Glycolysis/gluconeogenesis	Carbohidrate metabolism
507	GM	714	Gadph (383 aa) [zea mays]	8	gi|22240	41	7.21	39	6.18	Chloroplast	Glycolysis/gluconeogenesis	Carbohidrate metabolism
638	GM	788	Lactoylglutathione lyase [zea mays]*	15	gi|194701526	37	5.87	31	5.29	Chloroplast, peroxisome, plasma membrane, vacuole	Pyruvate metabolism	Carbohidrate metabolism
741	Non-GM	595	Lactoylglutathione lyase [zea mays]*	9	gi|194701526	37	5.87	31	5.54	Chloroplast, peroxisome, plasma membrane, vacuole	Pyruvate metabolism	Carbohidrate metabolism
589	Non-GM	781	Sedoheptulose bisphosphatase1 [zea mays]	11	gi|226506366	42	6.08	41	6.16	Cytosol	Calvin cycle	Carbohidrate metabolism
602	GM	797	Ferredoxin--nadp reductase, leaf isozyme [zea mays]	9	gi|226497434	41	8.53	33	5.84	Apoplast, chloroplast	Photophosphorylation	Energy metabolism
983	Non-GM	365	Thylakoid lumenal 19 kda protein [zea mays]	6	gi|226491484	27	5.48	19	4.19	Chloroplast	Photophosphorylation	Energy metabolism
794	GM	590	Adenine phosphoribosyltransferase [hordeum vulgare subsp. Vulgare]*	8	gi|194701624	19	5.14	22	5.08	Cytoplasm	Adenine metabolism (glycosyltransferase)	Genetic information processing
930	GM	335	Atp-dependent clp protease atp-binding subunit clpa [zea mays]	3	gi|226507418	25	9.39	16	6.47	Chloroplast	Proteolysis (nucleotide binding)	Genetic information processing
984	GM	449	Uncharacterized protein loc100194054 [zea mays]	7	gi|212721648	24	7.44	14	4.74	Not identified	Not identified	Not identified
412	GM	1279	Uncharacterized protein loc100383193 [zea mays]	17	gi|293336385	52	8.35	50	6.10	Not identified	Not identified	Not identified
739	Non-GM	325	Uncharacterized protein loc100502283 [zea mays]	5	gi|308080598	25	5.86	31	6.12	Not identified	Not identified	Not identified
789	GM	588	2-cys peroxiredoxin bas1 [zea mays]	8	gi|195626524	28	5.81	22	4.16	Apoplast, chloroplast	Antioxidant activity	Stress response
826	Non-GM	610	Apx1 - cytosolic ascorbate peroxidase [zea mays]	9	gi|226530305	27	5.55	27	5.92	Chloroplast, cell wall, plasma membrane	Antioxidant activity	Stress response
676	Non-GM	598	Glyoxylase1 [zea mays]	14	gi|162461576	32	5.59	35	5.87	Cytoplasm, nucleous	Gluthatione metabolism	Stress response
501	GM	701	Fructose-bisphosphate aldolase [Zea mays]*	10	gi|223975775	38	6.37	37	6.15	Cell wall, chloroplast, cytosol, mitochondrion, nucleolus, membrane	Glycolysis/gluconeogenesis	Carbohidrate metabolism
444	GM	548	Fructose-bisphosphate aldolase, cytoplasmic isozyme 1 [Zea mays]	6	gi|195612198	38	6.26	41	5.81	Cell wall, chloroplast, cytosol, mitochondrion, nucleolus, membrane	Glycolysis/gluconeogenesis	Carbohidrate metabolism
446	GM	883	Fructose-bisphosphate aldolase, cytoplasmic isozyme 1 [Zea mays]	9	gi|195612198	38	6.26	41	6.13	Cell wall, chloroplast, cytosol, mitochondrion, nucleolus, membrane	Glycolysis/gluconeogenesis	Carbohidrate metabolism
400	Non-GM	919	GADPH (383 AA) [Zea mays]	12	gi|22240	41	7.21	37	6.20	Chloroplast	Glycolysis/gluconeogenesis	Carbohidrate metabolism
427	GM	874	Glyceraldehyde-3-phosphate dehydrogenase B, chloroplastic-like [Brachypodium distachyon]*	13	gi|194688752	47	5.95	44	5.77	Chloroplast	Glycolysis/gluconeogenesis	Carbohidrate metabolism
555	GM	595	Lactoylglutathione lyase [Zea mays]*	8	gi|194701526	37	5.87	31	5.26	Chloroplast, peroxisome, plasma membrane, vacuole	Pyruvate metabolism	Carbohidrate metabolism
664	GM	368	H (+)-transporting ATP synthase [Zea mays]	7	gi|311237	20	4.35	24	4.37	Chloroplast	Photophosphorylation	Energy metabolism
656	2.1× Non-GM	947	Chaperonin [Zea mays]	13	gi|195623400	26	8.67	24	5.20	Apoplast, chloroplast, mitochondrion	Protein folding (chaperone)	Genetic information processing
286	Non-GM	424	S-adenosylmethionine synthetase 1 [Zea mays]*	8	gi|194689980	43	5.57	53	5.84	Cytoplasm	S-adenosylmethionine synthesis	Genetic information processing
320	Non-GM	429	Uncharacterized protein LOC100193051 [Zea mays]	5	gi|212723748	30	5.42	48	6.16	Not identified	Not identified	Not identified
515	Non-GM	500	Uncharacterized protein LOC100194161 [Zea mays]	4	gi|212721598	37	8.86	29	6.06	Not identified	Not identified	Not identified
690	2.6× GM	774	2-cys peroxiredoxin BAS1 [Zea mays]	14	gi|195626524	28	5.81	22	4.68	Chloroplast	Antioxidant activity	Stress response
618	GM	708	Apx2 - Cytosolic Ascorbate Peroxidase [Zea mays]*	10	gi|194707280	27	5.28	27	5.53	Chloroplast, cell wall, plasma membrane	Antioxidant activity	Stress response
557	Non-GM	326	IN2-1 protein [Zea mays]	3	gi|195612768	27	5.16	26	5.19	Chloroplast	Protein glutathionylation	Stress response
689	Non-GM	596	Pathogenesis-related protein 10 [Zea mays]	9	gi|226499304	17	5.13	18	5.21	Extracellular compartment	Defense response	Stress response
476	Non-GM	572	Peroxidase 42 precursor [Zea mays]*	8	gi|238011418	26	5.43	32	5.26	Extracellular compartment	Antioxidant activity	Stress response

^a^Protein spot abundance: statistically significant values at *P* < 0.05. These protein spots are given in yellow boxes on Figure 3. ^b^Protein name according to the best hit of MASCOT search against NCBI database. ^c^Functional protein classification according to the KEGG PATHWAY Database. Additional categories are included when necessary.

Functional classification of the identified proteins, carried out in accordance with the Gene Ontology Database, showed that they belonged to one out of three main functional categories: (a) carbohydrate and energy metabolism, (b) genetic information processing, and (c) stress response.

Within these functional categories, the carbohydrate metabolism group constituted a significant proportion for both proteomes from both experiments, although represented by different proteins (47% of all identified proteins) (Table 2).

The Campos Novos experiment presented eight proteins that were detectable only in the GM. The remaining eight proteins were absent in the GM (Figure 4a and 4b). The Chapecó experiment presented seven proteins exclusive to the GM plants and seven that were exclusive to the non-GM plants (Figure 4c and 4d). Two proteins showed quantitative differences between GM and non-GM hybrids in Chapecó. A chaperonin protein and a 2-cys peroxiredoxin BAS1 were up-regulated in non-GM and GM plants, respectively.

Five different proteins were identified in fourteen spots. The proteins are ascorbate peroxidase (spots 826 and 618), fructose-bisphosphate aldolase (spots 629, 501, 444 and 446), lactoylglutathione lyase (spots 638, 741 and 555) 2-cys peroxiredoxin BAS1 (spots 789 and 690) and a GAPDH (spots 507, 400 and 427). These proteins are considered to represent different protein isoforms resulting from posttranslational modifications which introduce changes of molecular weight (MW) and/or isoelectric point (pI).

### Proteins related to carbohydrate and energy metabolism

Maize leaves accumulate high levels of enzymes involved in carbohydrate and energy metabolism. Since 15 proteins were assigned to be involved in this category, we have grouped them according to their physiological function in order to make a more comprehensive discussion.

Consequently, five protein groups were assigned as participants in the following metabolic pathways: (a) Glycolysis and/or Gluconeogenesis, (b) Photophosphorylation (c) Calvin Cycle, (d) Pyruvate metabolism, and (e) Starch biosynthesis.

Three spots were identified as glyceraldehyde 3-phosphate dehydrogenase (GAPDH; spots 507, 427 and 400) and four spots were identified as fructose-bisphosphate aldolase (spots 501, 444, 446 and 629) proteins. These enzymes were present in both maize types (GM and non-GM) and in both growth locations. These proteins are considered to represent different molecular versions resulting, most likely, from post-translational modifications. It has earlier been reported that GAPDH was down-regulated in the leaf proteome of MON810 plants grown under farm conditions [6].

Three other proteins were grouped as participants in photophosphorylation reactions. These proteins are directly related to the energy metabolism, acting as oxidoreductases (ferredoxin--NADP reductase; exclusive to GM plants in Campos Novos), eletron transport (hydrogen transporting ATP synthase; exclusive to GM plants in Chapecó) and calcium ion binding (thylakoid lumenal 19 kDa protein; exclusive to non-GM plants in Campos Novos). It has earlier been reported that oxidoreductase-type proteins were frequently up- or down-regulated in other comparative studies using MON810 maize kernel proteomes [5].

Interestingly, sugar compounds levels have been also found to be altered in MON810 plants [16]. Barros et al. [6] reported considerable changes in levels of glucose, fructose and sucrose with 14.0, 7.0 and 1.8 fold increase in MON810 plants, respectively.

Indeed, mature maize leaves maintain several complex developmental processes that are extensively based on carbohydrate metabolism. The explanation for extensive carbohydrate metabolism is the heavy energy demand required for metabolic processes that occur during flowering and grain filling.

In addition, the underlying idea is that by overexpressing a certain gene or by expressing it in a constitutive way (e.g. a transgene) it would always have the intended effect on the phenotype. But increasing evidence supports the idea that sometimes strong and constitutive promoters (e.g. CaMV-35S) involve a high energetic cost and it could somehow yield a penalty in transgenic plants [17,18] and, in other cases, the beneficial effects of the transgene might be masked by pleiotropic effects.

### Stress response proteins

In the frame of our study, stress induced proteins were found present in unmodified as well as in GM proteomes, and they represented five groups of proteins.

We were able to detect proteins related to glutathione metabolism (glyoxylase1 and IN2-1), the protein family of peroxidases (peroxidase 42 precursor and APx1) and the pathogenesis-related protein (PR10); these were only detected in non-GM proteomes. Differences in expression levels of stress-related proteins when comparing GM and non-GM maize plants have also been observed in several other studies. Coll et al. 2010 [19] used a microarray platform to evaluate transcriptional differences between the maize leaves of MON810 and its non-GM counterpart under real agricultural conditions. The authors noticed that proteins related to the glutathione metabolism (the glutathione-S-transferase protein) were overexpressed in GM plants. In addition, Zolla et al. [5] found the glutathione peroxidase protein only present in MON810 maize grains. Those authors detected other stress-related proteins, two of which were overexpressed in GM plants (ferritin and dehydrin proteins) and another one exclusive to unmodified plants (late embryogenesis abundant protein). Another class of stress related proteins, the heat shock proteins, was also found down-regulated in MON810 plants [16].

In the present study it was also revealed that 2-cysteine peroxiredoxin BAS1 (2-CP) proteins are over-expressed in GM plants from both locations. These proteins are highly sensitive to inactivation by reactive oxygen species, whereas 2-CP detoxifies H_2_O_2_ under normal conditions as well as under oxidative stress.

Further explanations for this extensive activity of oxidative stress enzymes come from oxidative phosphorylation described in the previous section. Indeed, oxidative phosphorylation is the source of reactive hydrogen, a poisonous compound for plant tissues. Peroxidases, which are endowed with xenobiotic functions, are of great importance for eliminating H_2_O_2_ resulting from oxidative phosphorylation.

The presence of stress related protein in conventional plants reveals differential expression pattern that could be linked to quantitative and/or qualitative factors. These factors might be linked to variation in functional units of the biological systems; considering the number of protein species per gene as a result of alternative splicing, reading frame, and post-translational modifications, trafficking and interactions, as protein complexes, rather than individual proteins [20].

### Genetic information processing proteins

We have detected four proteins related to genetic information processing. Two of these were only present in GM plants from Campos Novos. This concerns the adenine phosphoribosyl transferase (APT), and the ATP-dependent Clp protease ATP-binding subunit ClpA (Clp-ClpA).

APT plays an important role as a key enzyme for the unique route of adenine salvage in plants. Therefore, APT is constitutively expressed at relatively low levels in all cells and is classified as a housekeeping enzyme to reflect this basic role in cellular biochemistry [21]. Basse [22] found that the transcription of ZmAPT2 in *Zea mays* were 3.1-fold up-regulated in leaf tumor tissue compared to control tissue of the same age. In a word, ZmAPT2 may play key roles in plant growth and development, and its detailed functions need to be further studied.

On the other hand, the members of Clp-ClpA protease family interact with specific substrates to exert unfoldase activity. Energy-dependent proteolysis of such enzymes plays a key role in prokaryotic and eukaryotic cells by regulating the availability of certain short-lived regulatory proteins, ensuring the proper stoichiometry for multi-protein complexes, and ridding the cell of abnormal proteins [23]. Zolla et al. [5] found ATP-dependent Clp protease proteolytic subunit ClpP to be down-regulated in GM plant kernels.

The remaining two proteins related to genetic information processing observed in the present work were found up-regulated in non-GM plants in Chapecó (chaperonin protein and S-adenosylmethionine synthetase 1).

Enzymes within the chaperonin protein family provide favorable conditions for the correct folding of other proteins, thus preventing aggregation. For example, the S-adenosyl methionine synthetase 1, also known as methionine adenosyl transferase (MAT), is an important enzyme participating in many cellular processes. It acts as a major methyl group donor in transmethylation of proteins, nucleic acids, polysaccharides and fatty acids. It is also an important effector in the regulation of the biosynthesis of threonine and methionine [24].

Barros et al. [6] found similar results when comparing two GM maize hybrids with a near-isogenic non-GM hybrid using transcriptomics, proteomics and metabolomics data. The authors observed the overexpression of MAT in non-GM maize kernels. Furthermore, in the same study it was found that methionine metabolite was also overexpressed in GM plants with Roundup Ready herbicide tolerance (Event NK603). Moreover, Zolla et al. [5] found methionine synthase to be down regulated in GM plants kernels.

It is interesting to note that many of these genetic information-processing proteins are directly related to gene expression control.

### “Omics” data, natural variation and GM safety evaluation

Many view the use of the “omics” methods for profiling classes of molecules as potentially valuable tools in risk assessment, but it is far from consensus on the usefulness and applicability of these techniques for biosafety purposes [4,11,12,25].

Central to these procedures are comparative analyses of the GM crops with appropriate non-GM comparators that have a history of safe use. Comparative compositional analysis is one component of a comprehensive risk assessment approach and guidelines can be found to be described elsewhere [26,27].

It has been already discussed the utility of profiling especially in cases where the most scientifically valid isogenic and conventional comparator would not grow, or not grow as well as, for example, stress tolerant transgenic crops under the relevant stress conditions [8]. In such cases, the physiology of the two organisms under comparison would not be sufficiently substantially equivalent for direct comparison, and the only way to detect unintended changes would be through a survey of their molecular components [28].

Over the last few years, a number of published studies have focused on the investigation of possible unintended effects of the transformation event and expression of transgenes in plants, many of them based on general “omics” technologies [9,29-31]. However, results of such studies are not consistent or coherent. This may be explained by use of hybrids with different genetic backgrounds and/or different growth conditions, as well as variations in the methods applied [32].

Therefore, the aim of the present study was to broaden the state of knowledge about the inherent natural variability in GM crop composition induced by genetic background and its modulation by the environment. To date, no other study was able to characterize differentially expressed proteins from field-grown GM maize under a Brazilian genetic background.

The evaluation of GM maize proteomes under different agroecosystem conditions resulted in the detection of several proteins related to a diverse range of physiological metabolic pathways that could be easily grouped into three major categories: the carbohydrate and energy metabolism, genetic information processing and stress response. Within these, around 60% of all detected proteins (19 spots) were assigned to participate in the same metabolic pathway in both locations. Nevertheless, many of these proteins have also been detected in other studies. The compilation of the findings from these studies together with the results obtained from the present work reveal protein families that are involved in similar metabolic pathways. It is interesting to note that each of these studies was performed with a different plant hybrid expressing the same transgene cassette but grown under distinct environmental conditions (Table 3).

**Table 3:** Number of differentially expressed proteins/transcripts according to their physiological functions and revealed by proteomic and transcriptomic analysis observed by other authors when evaluating genetically modified maize (MON810) under different growing conditions

**Study reference**	**Study experimental condition/location**	**Study sampling (tissue)**	**Physiological function of identified proteins/transcripts**			
			**Carbohydrate and energy metabolism**	**Genetic information processing**	**Stress response**	**Other proteins**
This study (Campos Novos)	Agricultural (Brazil)	Leaves	8	2	3	0
This study (Chapecó)	Agricultural (Brazil)	Leaves	7	2	5	0
Coll et al., 2008	In vitro	Leaves	5	2	2	7
Zolla et al., 2008	Growth chambers	Grains	25	9	4	0
Barros et al., 2010	Agricultural (South Africa)	Grains	1	3	0	8
Coll et al. 2010	Agricultural (Spain)	Leaves	4	3	1	9
Coll et al. 2011	Agricultural (Spain)	Grains	2	4	2	0
Balsamo et al., 2011	Growth chambers	Leaves	0	0	0	1

Note: Uncharacterized proteins are not accounted in this table. All protein physiological categories assignments were performed according to KEGG.

It is widely understood that major changes in the proteome profile of GM crops will be driven by genotypic, environmental (geographical and seasonal) and crop management influences (and combinations thereof) than by genetic engineering [19,33-35]. However, large aggregate changes in the quantities of many different molecules are expected, especially when comparing non-isogenic lines and examining across seasons and locations. The presence of a relevant difference unique to the GMO being evaluated is not dependent of the overall variation observed in particular environment × gene scenarios or breeding conditions [8].

Therefore, the development of comprehensive databases on gene, protein and metabolite profiling of “conventionally” grown crops and “conventionally” bred crops grown under a range of environmentally variable conditions will provide a highly desirable benchmark for the safety assessment of alternative production systems or breeding practices [4,11].

Nevertheless, the absence of global databases on outputs from “omics” analyses need not, in reality, inhibit the use of these technologies in risk assessment as long as they are applied with the stringency expected for peer-reviewed publications and on well-designed field experiments carried out with due diligence, with the inclusion of all the relevant and replicated controls [4].

The results presented here, which is typical to the proteome community in terms of methodological approach; highlight the need for robust experimental design that encompasses the appropriate application of statistical procedures in order to be able to determine the different sources of variation in the dataset. These will all need to be considered to ensure sufficient power to allow the detection of changes in expression [36].

Finally, the emergence of data standards and public repositories facilitate the sharing and reuse of results as well as allowing independent validation of the results [37]; and these should also be considered when assessing GM safety.

## Conclusions

In conclusion, our results show that the environment was the major source of influence to the expression of GM maize proteins. Protein differences were observed in MON810 and non-GM agronomic field-grown maize with Brazilian genetic background. Although the 2DE technology allows the analysis of a limited dataset, differentially accumulated proteins represented less than 3.1% analyzed spots. In agreement with previous proteomics results, some of them were variety and/or field specific. However, few proteins were assigned to both treatments. Such observation indicates that the genome changes in GM maize may have an impact on the gene expression, but with a significant environment modulation. Nonetheless, the detection of changes in protein profiles does not present a safety *issue per se*; therefore, further studies should be conducted in order to address the biological relevance and implications of such changes.

## Methods

### Plant material and field experiment

The cultivation of MON810 transgenic maize (Monsanto do Brasil Ltda.) has been approved in Brazil in 2008. MON810 contains a genomic insert of the *cry1Ab* gene from *Bacillus thuringiensis*. The expression product of this gene is the insecticide protein (Bt toxin) Cry1Ab. Transgenic single cross hybrid seeds P32R48YG (Pioneer Sementes Ltda.) widely used for silage were kindly provided by the company. The near-isogenic, non-transgenic hybrid P32R48 (Pioneer Sementes Ltda.) was purchased from local markets. Seeds were tested for the presence of MON810-derived *cry1Ab* insert and its expression product by PCR and immune strip test (Envirologix), respectively (data not shown). After the confirmation of MON810 event in GM seeds and the absence in its non-GM counterpart, these were used in the experiment. Single cross hybrid seeds are the progeny derived from the cross of a maternal endogamous line “A” with the paternal endogamous line “B”. This seed population is, therefore, supposed to have a high genetic similarity (all individuals are genotype AB).

Field experiments were performed at two maize production areas with different agroecosystems conditions from October 2009 to February 2010. One growth area was located in Campos Novos, Brazil (latitude 27° 24′06″S and longitude 51° 13′30″W) Cfb climate (marine temperate), clay soil and an average of 1000 m altitude. The second growth area was located in Chapecó, Brazil (latitude 27° 05′ 45″ S and longitude 52° 37′ 04″ O) Cfa climate (humid subtropical), clay soil and an average of 600 m altitude. The experimental field consisted in a 120 m^2^ area divided into three replicate blocks (4 rows wide for each hybrid grown side-by-side, 5 m long, row spacing 0.8 m). Plots were sown at a density of 80,000 plants/ha and treated following standard agricultural practices in the region. Both locations had the same management. Weeds were controlled with pre-emergence application of 3 l/ha of Roundup Ready (glyphosate acid 480 g/L, Monsanto do Brasil Ltda.) and with post-emergence application of 1.25 l/ha Herbimix SC (Atrazine 250 g/L, Buschle & Lepper S.A.) between the lines. No fungicide or insecticide was applied.

Maize leaves were collected at R0 stage (57 days after sowing) during anthesis. Sampling was performed during early morning in both locations. Around 1 g of material was collected from the third upper leaf, consisting of a 5 cm long tissue piece located in the mid portion from which the central vein was removed. Samples were weighed, immediately placed in cryogenic tubes into a liquid nitrogen container and stored in −80°C freezer. Plants were carefully checked for the absence of *Helicoverpa zea* and *Spodoptera frugiperda* (corn earworm and fall armyworm, respectively) and necrosis. Three biological replicates were randomly sampled per maize hybrid each grown in a different plot.

### Protein extraction

Samples were separately ground with liquid nitrogen in a mortar. Protein extraction was carried out according to Carpentier et al. [38], i.e. by phenol extraction and ammonium acetate in methanol precipitation. Pellets were resuspended in urea/thiourea buffer (2% v/v Triton X-100 (Sigma-Aldrich Corporation, St. Louis, USA), 2% v/v Pharmalyte (GE Healthcare), 5 mM PMSF, 7 M urea and 2 M thiourea). Protein quantification was performed by means of the copper-based method 2-D Quant Kit (GE Healthcare) and stored at 4°C.

### Two-dimensional IEF/SDS–PAGE and protein staining

The extracted proteins were separated by 2-DE as described by Weiss and Görg [39]. In the isoelectric focusing step (IEF), Immobiline™ DryStrip gels with 13 cm and linear pH range 4–7 (GE Healthcare) were used. Strips were previously rehydrated with 750 μg of total protein and rehydratation solution (7 M urea, 2 M thiourea, 2% w/v CHAPS, 0.5% v/v IPG buffer (GE Healthcare), 0.002% w/v bromophenol blue). Strips were then focused on an Ettan IPGPhor IEF system (GE Healthcare) and subsequently equilibrated for 30 min in slow agitation in a Tris–HCl solution (75 mM), pH 8.8, containing 2% w/v SDS, 29, 3% v/v glycerol, 6 M urea and 1% w/v dtt or 2.5% w/v iodocetamide . The strips were then placed on top of SDS-PAGE gels (12%, homogeneous) for the second dimension run using a Hoefer DALT system (GE Healthcare) according to manufacturer’s guidance.

### Quantitative analysis of maize proteomes

Each experiment (Campos Novos and Chapecó) consisted of 18 gels, in which three biological replicates were sampled per hybrid (GM and non-GM) and three technical replicates were performed per sample, in a total of 36 gels. This design allowed the performance of both biological and technical variation statistical analysis [40]. The six samples (three biological replicates of each hybrid) were run together to reduce technical variation due to differences in electrophoresis pattern. Proteins were visualized by CBB G-250 colloidal stain (MS compatible) as described by Candiano et al. [41] which increases the staining sensitivity to approximately 1 ng of protein. Each gel was scanned using ImageScanner™ III (GE Healthcare). Cross-comparisons among the different samples were performed using the software Image Master 2D Platinum version 7.0 (GE Healthcare).

After manual verification of spots, gels were matched according to hierarchical condition. Gels from different treatments were first internally matched and only spots that were present on at least three gels within the treatment (with low coefficient of variance <20%) were included in the analysis.

### Mass spectrometry analysis and physiological clustering of identified proteins

Gel spots were excised and sent to Tromsø University Proteomics Platform (Tromsø, Norway) for processing and analysis. These were subjected to in-gel reduction, alkylation, and tryptic digestion using 2–10 ng/μl trypsin (V511A; Promega) [42]. Peptide mixtures containing 0.1% formic acid were loaded onto a nanoACQUITY UltraPerformance LC (Waters), containing a 5-μm Symmetry C18 Trap column (180 μm × 20 mm; Waters) in front of a 1.7-μm BEH130 C18 analytical column (100 μm × 100 mm; Waters). Peptides were separated with a gradient of 5–95% acetonitrile, 0.1% formic acid, with a flow of 0.4 μl/min eluted to a Q-TOF Ultima mass spectrometer (Micromass/Waters). The samples were run in data dependent tandem ms mode. Peak lists were generated from MS/MS by the ProteinLynx Global server software (version 2.2; Waters). Protein identification was performed by searching the National Center for Biotechnology Information non-redundant database (NCBInr), using the Mascot program (http://matrixscience.com). The data were first searched for contaminants against NCBInr database version 20120623 “all entries” (18713758 sequences). We have also searched against NCBInr database with taxonomy restriction to include only the 1081598 Viridiplantae (green plants) sequences. The following parameters were adopted for database searches: complete carbamidomethylation of cysteines and partial oxidation of methionines, peptide mass tolerance ± 100 ppm, fragment mass tolerance ± 0.1 Da, missed cleavages 1 and significance threshold level (*P* < 0.05) for Mascot scores (−10 Log (*P*)). Even though high Mascot scores are obtained with values greater than 325, a combination of automated database search and manual interpretation of peptide fragmentation spectra was used to validate protein assignments. Molecular functions and cellular component of proteins were browsed against KEGG Database (http://www.genome.jp/kegg/).

### Statistical analysis

The main sources of variation in the 2-DE experiment dataset were evaluated by unsupervised multivariate PCA, using Euclidean distance for quantitative analysis. PCA was first applied to determine the proportion of the total proteomic variation that originates from differences between biological and technical repetitions. PCA analyses were performed by examining the correlation similarities between the observed measures. The spot volume was analyzed using covariance matrix (XLSTAT software version 2013).

The data used to perform the PCA analyses were spot volumes obtained from imaging analysis of 2-DE gels. However, a different gel-to-gel comparison within the proteomic software has been performed. In order to avoid bias in PCA analysis, we have matched all gel images against a unique arbitrary reference image.

For the 2-DE experiment, one-way ANOVA was used to investigate differences at individual protein levels. The calculations are based on normalized spot volume based on the total intensity of valid spots in a single gel. Differences at the level *P* < 0.05 were considered statistically significant. Statistical analyses were performed using Image Master 2D Platinum version 7.0 (GE Healthcare).

## Abbreviations

2-DE: Two-dimensional gel electrophoresis; CBB: Coomassie brilliant blue; CHAPS: 3-[(3-cholamidopropyl) dimethyl-ammonio]-1-propanesulfonate; DTT: Dithiothreitol; GAPDH: Glyceraldehyde-3-phosphate dehydrogenase; GMO: Genetically modified organisms; GM: Genetically modified; IEF: Isoelectric focusing; IPG: Immobilized pH gradient; MS/MS: Tandem mass spectrometry; MW: Molecular weight; pI: Isoelectric point; PCA: Principal component analysis; PCR: Polimerase chain reaction; PMF: Peptide mass fingerprinting; Q-TOF: Quadrupole time-of-flight; SDS-PAGE: Sodium dodecyl sulfate polyacrylamide gel electrophoresis.

## Competing interests

The authors declare that they have no competing interests.

## Authors’ contributions

SZA and RON designed the study. SZA, MPG, OW and RON were responsible for data analysis and interpretation and drafted the manuscript; SZA carried out the 2D gel analysis and excised the protein spots; MPG and OW were responsible for mass spectrometry analysis and manuscript editing. All authors read and approved the final manuscript.
